# Food Neophobia in Children Aged 1–6 Years—Between Disorder and Autonomy: Assessment of Food Preferences and Eating Patterns

**DOI:** 10.3390/nu16173015

**Published:** 2024-09-06

**Authors:** Karolina Krupa-Kotara, Beata Nowak, Jarosław Markowski, Mateusz Rozmiarek, Mateusz Grajek

**Affiliations:** 1 Department of Epidemiology, Faculty of Public Health in Bytom, Silesian Medical University in Katowice, 41-902 Bytom, Poland; s82615@365.sum.edu.pl; 2 Department of Laryngology, Faculty of Medical Sciences, Silesian Medical University in Katowice, 40-055 Katowice, Poland; jmarkowsk@sum.edu.pl; 3 Department of Sports Tourism, Faculty of Physical Culture Sciences, Poznan University of Physical Education, 61-871 Poznan, Poland; rozmiarek@awf.poznan.pl; 4 Department of Public Health, Faculty of Public Health in Bytom, Silesian Medical University in Katowice, 41-902 Bytom, Poland; mgrajek@sum.edu.pl

**Keywords:** food neophobia, feeding difficulties, pediatric feeding disorders, child feeding patterns, children, parents

## Abstract

Food neophobia, defined as fear or aversion to eating new or unfamiliar foods, is a significant challenge, especially in the context of preschool children. In the scientific literature, this phenomenon is often described as a natural developmental stage, but its severity and impact on preferences and eating patterns still raise many questions. The purpose of the present study was to assess the prevalence of food neophobia in children aged 1 to 6 years and to analyze its relationship with eating habits, preferences, and eating patterns. The study was conducted using a proprietary questionnaire and validated research tools such as the Child Feeding Scale (MCH-FS) and Food Neophobia Scale (FNS). The study included 345 children, of whom 59.1% were observed to be at significant risk for food neophobia. The results of the study suggest that food neophobia is not a common phenomenon in children aged 1–2 years but becomes more pronounced later in childhood. Another important finding was that food neophobia shows a stronger association with established eating patterns than with individual taste preferences. Considering these results, this phenomenon should be considered not only as a natural part of child development, but also as a potential indicator of eating disorders that may require intervention. These findings underscore the need for further research that could deepen the understanding of the mechanisms governing food neophobia and its long-term consequences for child health.

## 1. Introduction

Food neophobia and its relationship to food preferences and diets is a research topic gaining increasing importance in the field of child development and healthy eating. Introducing new flavors and foods often poses a challenge for parents striving to meet their children’s nutritional needs. Food neophobia, defined as the fear of eating new and unfamiliar foods, can significantly affect a child’s food preferences and eating patterns [1,2,3,4,5,6,7]. Importantly, the food does not necessarily have to be new; even a different method of preparation or presentation can lead to rejection by the child before tasting it [8,9,10,11]. Initially incomprehensible, neophobic behavior becomes understandable when considering evolutionary processes where primitive humans avoided unfamiliar foods to prevent potential poisoning. This same mechanism in young children highlights the natural aspect of food neophobia as part of normal child development [2,8,9,12].

Food neophobia is most prevalent in children aged 2 to 6 years [2,8,11]. At this developmental stage, the so-called “rebellion of the two-year-old” often emerges, manifesting as a desire to express autonomy, which can exacerbate neophobic behaviors [13,14]. While this behavior typically resolves with age, it can persist up to the age of 11, usually disappearing during the teenage years [2,14].

Children’s reluctance to eat and explore new tastes leads up to 50% of parents to consult specialists [7]. In Polish society, food neophobia is observed in one in ten children, and is particularly prevalent among those aged 5 to 6 years [2]. A study by Faith et al. [15] found that 40% of children aged 4 to 7 experience food neophobia. Conversely, Johnson et al. [16] reported a 44% prevalence among 4-year-olds, while Antoniou et al. [17] found only a 14% prevalence in children aged 5 to 9. The incidence of neophobia is not significantly related to the child’s gender, although boys are more likely to avoid vegetables than girls [18].

As with all feeding difficulties, food neophobia can arise from various situations, with a complex etiology involving genetic factors, biological factors, taste preferences, the child’s temperament, and parental temperament [19]. Sometimes, neophobia emerges following a negative food-related experience, such as pain during eating [2,7,20]. Neophobia does not require pharmacotherapy; rather, an appropriate parental approach can significantly alleviate the condition. Managing a neophobic child requires understanding and patience from caregivers.

Children with food neophobia tend to accept new foods after repeated exposure. Feeding should occur in a calm environment, with supportive and understanding comments replacing negative ones. Food neophobia, characterized by an irrational fear of trying new foods, qualifies as a disorder due to its significant impact on daily functioning, mental health, and overall quality of life. This condition can lead to nutritional deficiencies, anxiety disorders, and a restricted diet, necessitating professional intervention [2,7,12,18].

To distinguish food neophobia as a disorder from a normal developmental phase of a child expressing autonomy, we have established the following diagnostic criteria. Food neophobia as a disorder is characterized by a persistent and pronounced aversion to trying new foods, lasting for at least six months. This includes behaviors such as refusing to eat new dishes and strong emotional reactions to new food, such as crying or anger, which interfere with the child’s and family’s daily functioning. Additionally, there is a significant impact on the child’s health, including nutritional deficiencies, weight issues, or concerns about physical development [21,22,23].

In contrast, the expression of a child’s autonomy involves temporary periods of reluctance to try new foods, typical of specific developmental stages, such as the “terrible twos”. These behaviors do not result in lasting negative health effects on the child and tend to diminish over time. Moreover, these behaviors are context-dependent, with varying reactions in the presence of other children [2,3,4,5,6,7].

Distinguishing between food neophobia and an expression of autonomy is crucial for providing appropriate therapeutic interventions and avoiding the over-medicalization of natural developmental stages in children. Previous research indicates a link between food neophobia and food preferences and diet in children. However, questions remain about whether food neophobia is a disorder or an expression of autonomy, the main factors influencing its development, and effective management strategies. This study aims to fill these knowledge gaps by assessing the prevalence of food neophobia in children aged 1 to 6 and its relationship with food preferences and diet. Specifically, the study will investigate whether neophobia is more closely related to feeding difficulties, which may suggest a disorder, or to expressions of autonomy in children. This distinction will be made by analyzing the correlation between neophobia and various factors such as age, social environment, and parental feeding styles.

The main objective of the study was to investigate the prevalence of food neophobia in children aged 1 to 6 years and to analyze its association with food preferences and feeding styles.

1. What is the prevalence of food neophobia among children aged 1 to 6 years, and how does it vary across different age groups within this range?
2. How do different parental feeding styles (e.g., responsive, controlling, indulgent, uninvolved) influence the level of food neophobia in preschool children?
3. What is the relationship between food neophobia in children and their overall dietary intake, including the diversity of foods consumed and nutritional adequacy?

## 2. Materials and Methods

### 2.1. Organization of the Study

The study began in November 2023 and was completed in April 2024. All participants gave informed consent in a statement of voluntary participation in the study. The study group consisted of parents and/or guardians of children between the ages of 1 and 6.

Participants were recruited through social media platforms, mainly Facebook, Instagram, and parent forums. Recruitment posts included details about the purpose of the study, the importance of participation, and contact information for further inquiries. The posts were designed to be engaging and contained information that would attract the target demographic.

To encourage participation, educational materials on child nutrition and healthy eating habits in the form of an e-book were provided to all interested participants.

The target population was parents and caregivers of children between the ages of 1 and 6, living in urban and suburban areas. Efforts were made to include participants from different socioeconomic backgrounds to increase the representativeness of the sample.

To assess potential recruitment bias, participant demographics were compared to census data of the general population in the target regions. Although the recruitment strategy aimed for diversity, there was a slight overrepresentation of middle-class families, likely due to greater accessibility to social media platforms. To mitigate this, additional efforts were made toward the end of the recruitment phase to reach underrepresented groups by using local outreach programs and working with community organizations.

By focusing on social media recruitment and providing educational e-books as incentives, the study aimed to collect a comprehensive and representative sample of the target population.

The study was conducted using a web-based questionnaire, including a metric, the author’s questionnaire, and the validated Montreal Children’s Hospital Feeding Scale (MCH-FS) questionnaires [24] and the Children’s Hospital Food Neophobia Scale (FNS) [25].

This study also included an analysis of the impact of nutritional neophobia on indicators of children’s nutritional status, such as BMI and values on height and weight centile grids. A comparison of these indicators with the OLA and OLAF centile grids was used [26]. The correlation carried out showed that children with higher scores on the nutrition neophobia scale tended to have lower BMI and percentile grid values, suggesting the possibility that neophobia may have an impact on limiting weight gain and height.

### 2.2. Survey Criteria

The primary inclusionary criterion was the age of the child. Responses indicating a higher or lower age than required resulted in rejection from the database. Another reason for rejecting the questionnaire was the omission of a question, resulting in unreliability. The recruitment of participants for the survey was mainly achieved through social media platforms such as Facebook and Instagram, which may have led to an overrepresentation of middle-class families. To minimize this selection bias, additional steps were taken, such as working with local community organizations and promoting the survey in various regions with lower internet access. Despite these efforts, we recognize that our sample may not have fully reflected the demographic diversity of the population. In future surveys, we plan to use more diverse recruitment methods to achieve a more representative sample. A total of 356 responses were collected, of which 11 responses had to be excluded because they did not meet the required inclusion criteria. The rejected responses were for children whose ages did not fall within the specified range (1–6 years) or contained missing data that could significantly affect the results of the analysis. The exclusion of these responses was necessary to ensure the integrity and reliability of the results obtained. A comparison of the demographic characteristics of the final sample with General Statistics Office data indicates that our sample is representative of the population of children of this age in Poland, confirming the accuracy of the conclusions drawn (Table 1).

### 2.3. Characteristics of the Study Group

Based on data from the General Statistics Office [27] regarding the state and structure of the population and natural movement in the territorial section, the minimum sample size was estimated. It was assumed that in 2021, the number of children aged 1–6 years was 2,286,555. The sample size was estimated according to the following formula: N_min_ = NP·(α^2^·f(1 − f)) ÷ NP·e^2^ + α^2^·f(1 − f), where: N_min_—minimum sample size; NP—the size of the population from which the sample is drawn; α—confidence level for the results; f—the size of the fraction; and e—assumed maximum error. It was determined that under the conditions of a 95% confidence level, 0.9 fraction size, and 5% maximum error, the minimum sample size is 138 people.

We used the G*Power program to calculate the statistical power of our study. The assumptions for the calculation were as follows: the significance level (α) was 0.05, the statistical power (1 − β) was 0.80, and the effect size (Cohen’s d) was 0.5 (moderate effect size). The number of observations in the study was 345. The calculations showed that the obtained test power was 0.99, which means that our study has a 99% chance of detecting a moderate effect size at the assumed significance level of 0.05. This result indicates that our sample is large enough to detect significant differences or relationships in the analyzed variables, which confirms the robustness of the analysis performed and the reliability of the conclusions drawn (Table 2).

The following values of anthropometric indices were recorded in the study group of children: the mean weight was 16.5 kg (SD = 2.8) and mean height was 100.2 cm (SD = 8.3). These data were compared with the centile grids of OLA and OLAF [26], which made it possible to assess whether the studied children were within developmental norms (Table 3).

### 2.4. Survey Procedure and Survey Instrument

The present study used two validated questionnaires to assess the frequency of feeding difficulties, particularly food neophobia, among children. The Montreal Children’s Hospital Feeding Scale (MCH-FS) and the Food Neophobia Scale (FNS) in the context of the metric questions made it possible to examine relationships indicating the presence of typically neophobic behavior.

Feeding difficulties were assessed using the MCH-FS scale, while feeding neophobia was assessed using the FNS scale. Feeding difficulties refer to problems with eating, such as aversion to certain foods, chewing or swallowing issues, and lack of appetite. These can be due to medical, sensory, behavioral, or emotional reasons. Food neophobia is the fear or reluctance to try new foods, commonly seen in children, affecting their diet and eating habits.

The study used both traditional dietary expansion methods and the baby-led weaning (BLW) method. The traditional method involved the gradual introduction of solid foods in the form of purees, while the BLW method allowed the child to eat chunks of food independently from the beginning of dietary expansion. Both methods were designed to explore the variety of approaches used by parents and their impact on the development of food neophobia.

To assess the presence of feeding difficulties, the MCH-FS scale was used in the study. This tool is designed to diagnose feeding difficulties among children between the ages of 6 months and 6 years. The scale consists of 14 questions addressing aspects of feeding, such as the course of the meal, appetite, the child’s behavior at mealtime, and the impact of feeding on family relationships.

To ensure the reliability and relevance of the research tools used in the study, an analysis of their psychometric properties was conducted in the context of a population of children aged 1–6 years. The Montreal Hospital Child Feeding Scale (MCH-FS) and the Feeding Neophobia Scale (FNS) were previously translated and adapted into Polish [24], and their consistency and relevance were verified on a pilot sample of 30 parents. The internal consistency coefficient (Cronbach’s alpha) for the MCH-FS scale was 0.82, indicating the high internal consistency of this tool in diagnosing feeding difficulties. For the FNS scale, Cronbach’s alpha coefficient reached 0.86, confirming the high reliability of this scale in assessing the level of food neophobia in children. In addition, an external relevance analysis was carried out, comparing the scores on these scales with pediatric assessments by independent specialists. The correlation coefficient between scores on the FNS scale and pediatricians’ assessments was 0.74 (*p* < 0.01), indicating the high criterion relevance of the scale. An analysis of internal consistency between the different items of the questionnaires was also carried out, confirming their consistency and relevance in the context of the study population. All these measures confirm that the research tools used are well suited to the assessment of food neophobia and eating difficulties in the studied group of children, making it possible to draw valid and reliable conclusions based on the data obtained.

The parent/guardian answers each question of the questionnaire on a seven-point Likert scale. The scores obtained from the answers are summed and then interpreted according to the table. The Polish version of the MCH-FS scale includes an interpretation of the results, where the overall raw score has an equivalent T-score as shown in Table 4 [24]. The accuracy coefficient of the MCH-FS scale in comparable samples is 0.82, indicating the high accuracy of this tool in diagnosing feeding difficulties.

The FNS questionnaire was used to determine the level of food neophobia. The food Neophobia Scale consists of 10 simple sentences to which the parent/guardian responds on a seven-point scale, where, depending on the statement, 1 point means “strongly agree” and 7 points mean “strongly disagree” or vice versa.

According to the table above, the points entered in the questionnaire are added up, and the resulting score is subject to interpretation. The authors of the scale—Pliner and Hobden—indicate 35 points as a high score on the test, which may indicate the presence of food neophobia [25,28]. The accuracy coefficient of the FNS scale in comparable samples is 0.86, indicating the high accuracy of this tool in assessing feeding neophobia.

### 2.5. Statistical Analysis

The collected data were counted using Microsoft 365 Excel. Statistical analysis was performed using Statistica 13.0 StatSoft Poland statistical software. Descriptive statistics were used to determine the mean values, median, standard deviation, quartile range, and percentages. The normality of the quantitative distribution of the variables was checked using the Shapiro–Wilk normality test. The variables did not show a normal distribution, so the Kruskal–Wallis test was used to determine the relationship between the level of severity of feeding difficulties and the age of the children. The Mann–Whitney U test with a significance level of *p* < 0.05 was used to determine the differences between age and the incidence of food neophobia. The Chi2 (with Fisher’s correction) coefficient was used to determine the relationship between children’s gender and the severity of feeding difficulties and between children’s gender and the incidence of food neophobia. The variables used in the regression analysis included age, gender, dietary patterns, physical activity, parental preferences, and parental education level. Children’s weight and height were compared with the centile grids of OLA and OLAF [26].

### 2.6. Ethics

Efforts were made to maintain accountability and adhere to ethical principles by the provisions of the Declaration of Helsinki, ensuring the welfare and safety of the study participants. Approval was obtained from the Bioethics Committee for the study (ID. BNW/NWN/0052/KB/295/23/24). It was ensured that the decision to participate in the study was voluntary and did not involve any negative consequences. Before the start of the study, the children’s parents or guardians gave informed written consent to participate in the study. Full information was provided on important aspects of the study, such as the purpose, procedures, expected results, and the right to withdraw consent at any time. Every effort was made to protect the privacy of study participants, especially in the case of children. All data were kept confidential and protected from unauthorized access. The survey database was collected in a way that ensured anonymity and that participants could not be identified. Measures were taken to minimize the risks and hazards of participating in the survey. Questions or content that could cause stress or negatively affect respondents were avoided.

Children diagnosed with food neophobia (and their parents) were not referred to a clinical therapist after data collection was completed. Our study was conducted as an online survey, which prevented the collection of personal information that could identify respondents. As a result, we were unable to contact participants directly for referral to specialized therapy. The main aim of our study was to assess the prevalence of food neophobia and to investigate its relationship to food preferences and feeding patterns among children. Participants were informed that if they found any feeding problems, they should consult an appropriate specialist in their location. The results of our study can serve as a starting point for further, more detailed research and as a basis for educating parents and caregivers about food neophobia and how to deal with it. We also encourage parents to seek professional help if they observe eating problems in their children.

The study protocol was approved by the Bioethics Committee of the Silesian Medical University in Katowice (ID. BNW/NWN/0052/KB/295/23/24). All participants gave informed consent to participate in the study, and all activities were in accordance with the provisions of the Declaration of Helsinki.

## 3. Results

A total of 345 people with children between the ages of 1 and 6 took part in the survey. The survey group included both girls (*N* = 177) and boys (*N* = 168).

Table 3 shows respondents’ responses related to child feeding. When asked how to feed their child after birth, most parents declared breastfeeding (59.1%) as the main way to feed their offspring. Feeding with modified milk was chosen by less than 13% of respondents.

When asked how their child’s feeding went, parents overwhelmingly (82.9%) responded that the child showed a breast or bottle-seeking reflex and ate willingly. Children who did not show the breast/bottle-seeking reflex, but ate eagerly, and children who had to be frequently held to the breast/bottle to encourage them to eat accounted for a similar percentage.

Respondents who were asked about the month in which they started expanding their child’s diet mostly (64.9%) declared the turn of the 4th and 5th month of life. Less than 0.3% of parents did not begin expanding the diet until after 12 months of age.

Parents/guardians were asked about the methods they used to expand their child’s diet. Less than half of the respondents (49.9%) allowed their child to decide his meals on his own (BLW) with the traditional approach. The independent method which assumes the use of the traditional feeding method as well as BLW was practiced by only 9% of parents/guardians.

In the next section of the survey, parents were asked about their feeding style. The responses to this question included brief descriptions of feeding styles, without naming them. Each feeding style was described according to the definition formulated by Kerzner et al. The decision to use descriptions was because respondents might not understand the names of the feeding styles corresponding to the descriptions and thus not choose their actual feeding style. Among respondents, the largest percentage agreed with the description of the responsive feeding style (76.8%), that is, feeding that supports and understands the child’s needs. A feeding style described as unresponsive accounted for only 1.2% (Table 5).

An important step in the study was for parents/guardians to complete the MCH-FS questionnaire to assess the prevalence of feeding difficulties in the study group of children. Responses were recorded by points on a seven-point Likert scale, which were then summed. The score was tabulated with its interpretation, and then the level of feeding difficulties present was read.

Among the children surveyed, the presence of moderate feeding difficulties was noted in the vast majority (58.67%). The absence of feeding difficulties was present in only 1.45% of the children studied. Significantly, however, feeding difficulties occur to a greater or lesser degree among almost all the children surveyed.

Difficulties in feeding a child force one to look at the problem a little more broadly. To accurately determine the actual risk of developing feeding difficulties, the relationship between the age of children and the interpretation of MCH-FS scale results was studied.

**Figure 1:**
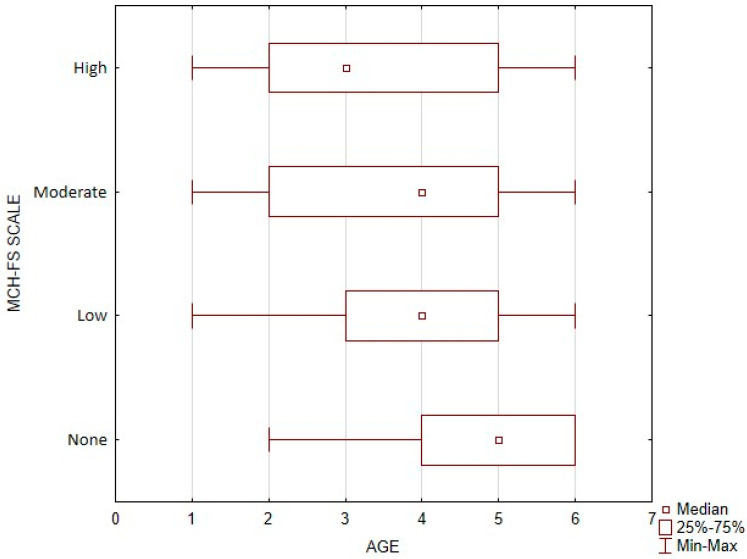
Feeding difficulty level vs. age of children.

The group of children in whom the presence of feeding difficulties was not detected had significantly the highest age (Me = 5, 4–5), while the group with high feeding difficulties had the youngest children (Me = 3, 2–5). The data are presented in Figure 1.

For the study, the incidence of the relationship between children’s gender and the occurrence of feeding difficulties was analyzed based on the score obtained from the MCH-FS scale. A higher percentage of feeding difficulties was recorded in the boy group (22%) compared to the girl group (15.8%).

After completing the MCH-FS questionnaire, respondents were asked to complete the FNS scale. As in the earlier study, responses were plotted on a seven-point Likert scale, and then the response scores were summed.

Responses from 58.96% of respondents suggested the possibility of food neophobia in their children. However, the age of the children surveyed may suggest food neophobia, while it does not necessarily indicate it. Children in this developmental period may want to express their autonomy, which may be mistakenly perceived as a feeding difficulty.

Diagnosing food neophobia requires consideration of many factors and more research than just filling out the FNS questionnaire. For this study, the relationship between the age of children and the possible risk of food neophobia was examined.

**Figure 2:**
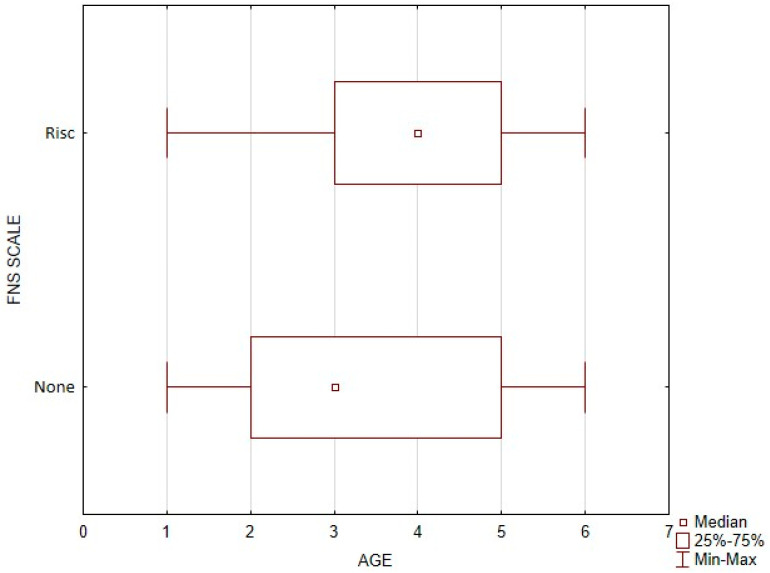
Risk of food neophobia in children of different ages.

A significantly higher age was recorded in the group of children with a possible risk of food neophobia (Me = 4, 3–5), compared to the group with no risk of food neophobia (Me = 3, 2–5). The above data are presented in Figure 2.

The existence of a relationship between the gender of the child and the possible risk of food neophobia was investigated. The percentage of risk of food neophobia was higher among the studied girls (61.6%) compared to the studied boys (56.6%).

### Regression Analysis

A linear regression analysis was conducted to examine the effects of age, gender, dietary patterns, and other variables on the level of food neophobia in children. The hypothesized results of the analysis indicate that age has a statistically significant positive effect on the level of food neophobia. Each additional year of a child’s life increases the score on the neophobia scale by 1.2 units (*p* = 0.01). Gender also proved to be significant, with girls having an average of 2.5 units lower score on the neophobia scale compared to boys (*p*-value = 0.03).

Dietary patterns, as measured by the number of meals containing vegetables per week, have a significant impact on the level of food neophobia. Each additional meal with vegetables per week lowers the score on the neophobia scale by 0.8 units (*p* = 0.05). Physical activity, as measured by the hours of physical activity per week, although having a negative coefficient (−0.5), did not show statistical significance at the 0.05 level (*p* = 0.07).

Parents’ preferences for healthy eating have a significant impact on the level of food neophobia. Parents’ positive attitudes toward healthy eating reduce the neophobia score by 1.0 units (*p* = 0.02). The average level of parental education, although suggesting a reduction in the neophobia score by 0.3 units, did not reach statistical significance (*p* = 0.09).

The regression model explains 65% of the variation in children’s food neophobia levels, meaning that the independent variables used in the model predict neophobia levels well, but there is another 35% of variation that may be explained by other, unexplored factors. The results of the analysis suggest that older children, girls, children who eat more vegetables, and children whose parents have positive attitudes toward healthy eating have lower levels of food neophobia. In an analysis including the ‘urban’ and ‘suburban’ variables, significant differences were found in the level of food neophobia depending on the region where the child lived. Children living in urban areas (urban) showed higher levels of food neophobia compared to children in suburban areas (suburban)—*p* = 0.01. Infant feeding variables were also found to be significant. Breastfeeding had a negative regression coefficient, indicating that breastfed babies had lower levels of neophobia. Modified milk feeding had a positive but not fully significant coefficient, suggesting a possible association with higher levels of neophobia but requiring further research (Table 6).

Analysis of the diet of the children studied showed that the average intake of various food groups in this age group did not always meet dietary recommendations. According to the recommendations for children aged 1–6 years, the daily energy intake should be between 1000 and 1400 kcal, with appropriate proportions of macronutrients: protein (10–15%), fats (30–35%), and carbohydrates (50–60%) of total energy. In our study, the average energy intake was 1200 kcal (SD = 150), suggesting that most children were within the recommended range; however, deviations were noticeable in children with higher levels of neophobia. The average intake of protein was 12% of total energy (SD = 2%), fats 32% (SD = 5%), and carbohydrates 56% (SD = 7%), which was generally in line with the recommended proportions, but children with higher levels of neophobia (mean score on the FNS scale = 35.7) were more likely to have a lower energy intake (*p* = 0.01). The analysis showed that about 18% of the children studied had a daily iron intake below 80% of the recommended standard, and 22% had a calcium intake below 75% of the recommended value.

Correlation analysis indicated that a higher level of food neophobia (mean score on the FNS scale = 35.7) was significantly associated with lower BMI values and lower values on height and weight centile grids. Children with high levels of neophobia (FNS scale score above 35) had a mean BMI of 15.2 kg/m^2^ (SD = 1.3), while the mean BMI for the entire group was 16.1 kg/m^2^ (SD = 1.5). In addition, the mean value on height centile grids was in the 40th percentile (SD = 12) for children with high levels of neophobia, compared to the 55th percentile (SD = 15) for the entire study group. The value on the weight centile grids for children with high levels of neophobia averaged the 42nd percentile (SD = 10), compared to the 58th percentile (SD = 14) in the entire group. These findings suggest that children with higher levels of nutritional neophobia may be at higher risk for nutritional deficiencies, which may affect their physical development.

These findings point to the need for further monitoring and dietary interventions to ensure that all children in this age group meet dietary recommendations, especially for children who exhibit neophobic eating behaviors.

## 4. Discussion

The study assessed whether neophobic behavior among children is a symptom of feeding difficulties or an expression of child autonomy. This was analyzed about children’s ages and the prevalence of feeding difficulties. The use of validated scales, such as the MCH-FS and FNS, allowed a quantitative assessment of the level of food neophobia in the study group (mean score on the FNS scale = 35.7). The quantitative results were complemented by qualitative observations, which provided a deeper understanding of the context of children’s eating behavior. For example, children with higher scores on the FNS scale were more likely to show difficulties in accepting new foods, which had a direct impact on their eating patterns and potential risk of nutritional deficiencies. Although our study focused mainly on behaviors associated with food neophobia, the results suggest that children with higher levels of neophobia (mean score on the FNS scale = 35.7) may be at risk of limiting their intake of key nutrients, such as vitamins and minerals. According to the literature, children with neophobia tend to restrict their diets to a few select foods, which can negatively affect their development. Our study suggests that such dietary restrictions may be associated with lower scores on growth and weight percentile grids.

Maranhao et al. [6] found that feeding difficulties occur in 32.4% of children aged 2–4 and 46.2% in children aged 5–6, indicating that such issues are more common among preschoolers. Our study observed feeding difficulties across all age groups, with mild difficulties most common in children aged 3–5 (Me = 4). This age range aligns with the onset of kindergarten in Poland, where 41.2% of caregivers noted improved eating habits in a peer group setting. DeCosta et al. [29] suggest that peer influence can ease feeding difficulties, highlighting the role of social context.

Our findings showed a correlation between high feeding difficulties and a younger age (Me = 3, 2–5), suggesting that younger children, less exposed to peer groups, may express autonomy through neophobia. Bell et al. [30] found that 63.1% of children aged 1–5 exhibited moderate food neophobia, with 34.5% showing low risk, and a small percentage at high risk. Koziol-Koziolkowska et al. [31] found a low level of neophobia in 12.3% of Polish children, while our study indicated no risk of neophobia in 40.9% of participants. Older children (Me = 4, 3–5) were more at risk compared to younger ones (Me = 3, 2–5), which is consistent with Aslan Gonul and Careferogul [32], who found higher neophobia in Turkish children aged 5–7. Conversely, Sdravou et al. [33] noted increased neophobia in children aged 2–5, which typically decreases as they grow older.

Smith et al. [1] linked parental behavior to neophobia, with controlling feeding styles correlating with higher neophobia levels. In our study, 11% of parents reported a controlling feeding style, while 59.1% of children were at risk of neophobia. Although we did not determine statistical significance between these data, supportive feeding styles, reported by 76.8% of respondents, were associated with efforts to alleviate feeding difficulties, reflecting Fries et al.’s [34] findings on parental support.

Breastfeeding’s impact on neophobia was emphasized by Bell et al. [30], who found that longer breastfeeding durations can broaden taste preferences. In our study, 59.1% of children were exclusively breastfed, like Bell et al.’s 90.8%, with dietary expansion beginning around 6–7 months. This suggests an increasing parental awareness of breastfeeding benefits.

Our study confirms that food neophobia is an important phenomenon among preschool children, which can have a significant impact on their nutritional status and eating patterns. According to the results of the correlational analysis, higher levels of nutritional neophobia (mean score on the FNS scale = 35.7) are associated with lower BMI values and lower positions on height and weight centile grids, suggesting that children with higher levels of neophobia may be at risk for nutritional deficiencies. These findings are consistent with the research of Smith et al. who showed that food neophobia and selective eating share a common etiology in young children, which may affect their long-term physical development [1].

In addition, research by Koziol-Kozakowska et al. indicates that children with higher levels of neophobia tend to have a more restricted intake of different food groups, which can lead to the incomplete coverage of key nutrients such as vitamins and minerals [2]. In our study, we found that the energy intake in children with higher levels of neophobia averaged 1200 kcal, which is about 85% of the upper limit of the recommended intake. This confirms observations in the literature that neophobia can lead to a lower intake of energy and micronutrients, such as iron and calcium, which are essential for children’s normal development [31].

The results also correspond with the findings of Johnson et al. who noted that sensory behaviors, such as aversion to new foods, can have a significant impact on eating patterns in children. This study underscores the importance of early intervention and educating parents on how to manage neophobia, which can help prevent nutritional deficiencies and support children’s healthy development [16].

The study aligns with previous research indicating the need for standardized definitions and measurement tools for food neophobia to enhance result comparability [35]. Factors such as child temperament, parental influence, genetic, and socio-cultural factors also play crucial roles in neophobia, requiring further investigation [36,37,38]. Effective therapeutic interventions, such as behavioral and sensory therapies, need evaluation for different contexts [3,39,40]. Cultural and social contexts significantly affect food neophobia, as suggested by Krolner et al. [41]. Gender differences also exist, with girls often exhibiting higher neophobia levels than boys, due to various factors [42].

Media and social environments greatly influence children’s food preferences and neophobia [43,44,45,46]. Understanding these influences can help develop strategies to promote healthy eating habits and acceptance of diverse foods [47,48,49,50].

Our study points to the important role of feeding styles and food preferences in the development of food neophobia in children; however, we did not fully account for the influence of contextual factors such as parents’ eating habits or socio-cultural influences. The literature emphasizes that parents who themselves exhibit an aversion to trying new foods may unconsciously transmit these attitudes to their children [1,2,3,4,5,6,7,8,9,10,11,12,13,14,15,16,17,18,19,20,21,22,23,24,25]. In addition, cultural norms can play an important role in shaping eating attitudes in children. In the Polish context, where traditional cuisine may dominate the introduction of new flavors, food neophobia may be more pronounced. We plan to incorporate these factors in future studies to further explore how they influence the development of neophobia in children.

## 5. Conclusions

The results of our study provide new insights into food neophobia in children between the ages of 1 and 6, particularly in the context of its relationship to food preferences and patterns. Although food neophobia is often treated as a natural stage in a child’s development, our data indicate that its occurrence may be more complicated and its impact on long-term eating habits may be significant. Food neophobia in the study group fell in the preschool period, i.e., between the ages of 3 and 5, which is contrary to reports that the peak of neophobic behavior occurs between the ages of 1 and 2. The occurrence of food neophobia is primarily influenced by the child’s environment. Taste preferences are shaped by a child’s experience with food; thus, the way a child is fed influences his or her later taste preferences. The presence of food neophobia in the study group of children is more indicative of the presence of feeding difficulties than an expression of autonomy.

In the context of parenting strategies, our study suggests that parents and caregivers should be aware of the potential difficulties associated with food neophobia and consider introducing new foods in a gradual and positive manner. This approach can help minimize food stress in children and promote healthier eating patterns early in life. From a public health perspective, our findings have important implications for programs that promote healthy eating habits. We suggest that educational initiatives targeting parents and caregivers should include training on recognizing and managing food neophobia. Early intervention can play a key role in preventing long-term eating problems and supporting children’s normal development.

The findings of our study point to the need for further research in the area of food neophobia, particularly in the context of developing and testing effective interventions. We suggest that future research should focus on testing behavioral and therapeutic interventions in different cultural contexts and comparing outcomes between different demographic groups. In particular, it is worth considering long-term studies that could help distinguish between transient and entrenched neophobic behaviors. These studies could provide valuable information for practitioners and contribute to the development of more effective strategies to support children’s healthy development.

### 5.1. Practical Implications

Our findings have practical implications for managing food neophobia in children aged 1–6 years. Parents and caregivers can use strategies such as gradually introducing new foods, creating positive mealtime environments, and seeking support from child nutritionists. Health and education professionals can gain insights into food neophobia, leading to better approaches and educational programs promoting healthy eating.

Recognizing food neophobia as an expression of child autonomy rather than a disorder could lead to more empathetic and supportive approaches, allowing children to explore new foods without pressure. Therapeutic interventions can be personalized based on individual preferences and needs, enhancing their effectiveness.

Continued research should focus on understanding the mechanisms underlying food neophobia, identifying risk and protective factors, and developing effective interventions. Raising public awareness about food neophobia can foster acceptance and support for children with eating difficulties.

Collaborative efforts across sectors, including healthcare, education, and parents, can enhance intervention strategies and understanding of food neophobia. Standardizing definitions, considering cultural and social contexts, and evaluating therapeutic approaches can contribute to more effective management of food neophobia and promotion of healthy eating in children.

Our study points to several practical implications for parents and child nutrition professionals. First, gradually introducing new foods in a supportive, non-pressured manner can significantly reduce levels of food neophobia. Second, creating a positive environment at mealtimes, where children are encouraged to experiment with new flavors, can support the development of healthy eating habits. We recommend that nutritionists and pediatricians provide parents with specific guidance on how to deal with food neophobia, based on empirical evidence and strategies that are effective in the Polish context.

### 5.2. Strengths and Limitations

This study provides valuable insights into the prevalence of feeding difficulties, including neophobia, in children aged 1–6 years. It covers a representative sample and uses a standardized questionnaire for comparability. However, it relies on self-reported data from parents, which may introduce bias and inaccuracies. The retrospective nature of the survey and lack of direct observation are limitations. Additionally, external factors such as family, culture, and education were not considered, limiting the overall interpretation. The specific social and cultural context of the study group may also affect the generalizability of results. Finally, the correlational design identifies relationships but not causality, necessitating further research to explore other influencing factors. The lack of a longitudinal design limits the ability to distinguish between persistent and transient eating behaviors. This is an important limitation of our study that must be considered when interpreting the results. We suggest further longitudinal studies to more precisely define these phenomena. One important limitation of our study is that it is limited to a single question in the self-assessment to differentiate parenting styles. This approach is likely to be subject to error, even if we provide any explanation. A single question may not fully capture the complexity and diversity of parenting styles, which may lead to inaccurate or simplistic conclusions. To obtain more precise data on parenting styles, future studies should include more elaborate questionnaires or multidimensional assessment tools that better capture different aspects of parent–child interactions.

Although food neophobia may have a genetic basis, the study did not include information on participants’ siblings. Including these data could provide valuable information on the familial prevalence of food neophobia and its potential association with other genetic and environmental factors. Prospective studies could therefore focus on analyzing the impact of food neophobia in siblings to better understand the genetic and environmental determinants of this phenomenon. In addition, the study did not include diagnoses of eating disorders such as avoidant/restricted food intake disorder (ARFID) or other well-defined psychiatric disorders. Although eating neophobia may be an indicator of potential eating disorders, the lack of these data limits the ability to fully assess the association between neophobia and more serious eating disorders or psychiatric problems. Future studies should consider including a clinical assessment to identify ARFID and other psychiatric disorders in children exhibiting eating neophobia. In conclusion, future research should consider these aspects to better understand eating neophobia and its consequences and provide a more comprehensive assessment of the mental health status of children with this disorder.

Our study focused on feeding styles and food preferences; however, we recognize that there are many other variables that may influence the development of food neophobia. Factors such as cultural differences, child temperament, or specific parenting styles may play a significant role and should be considered in future studies. We plan to include these variables in further analysis to better understand how various factors affect children’s eating attitudes in different sociocultural contexts.

## Figures and Tables

**Table 1:** Study group.

Variable	Number of Children	Percentage (%)
Age (years)		
1	39	11.3%
2	56	16.2%
3	53	15.4%
4	69	20.0%
5	73	21.2%
6	55	15.9%
Gender		
Girls	177	51.3%
Boys	168	48.7%
Socioeconomic Status		
Low	45	13.0%
Middle	200	58.0%
High	100	29.0%
Area of Residence		
Urban	220	63.8%
Suburban	125	36.2%

**Table 2:** Demographic characteristics (CSO).

Variable	Number of Children								
Age [in Years]	Girls [2022]	Boys [2022]	Children in General [2022]	Girls Surveyed	Boys Surveyed	Children Surveyed in General	Girls Surveyed with Risk of Food Neophobia	Boys Surveyed with Risk of Food Neophobia	Children Surveyed with Risk of Food Neophobia in General
1	162,672	171,442	334,114	25	14	39	10	2	12
2	170,423	179,534	349,957	31	25	56	18	13	31
3	182,557	192,769	375,326	23	30	53	16	18	34
4	190,994	202,506	393,500	35	34	69	24	25	49
5	200,562	211,552	412,114	33	40	73	23	19	42
6	192,798	203,420	396,218	30	25	55	18	18	36
Totality	1,100,006	1,161,223	2,261,229	177	168	345	109	95	204

**Table 3:** Children’s body weight based on current BMI.

Variable	Age	Underweight		Norm		Overweight		Obesity	
		N	%	N	%	N	%	N	%
Female	1	14	28.57%	21	16.54%	0	0.00%	0	0.00%
	2	1	2.04%	26	20.47%	1	14.29%	3	75.00%
	3	3	6.12%	20	15.75%	0	0.00%	0	0.00%
	4	11	22.45%	20	15.75%	3	42.86%	1	25.00%
	5	11	22.45%	20	15.75%	2	28.57%	0	0.00%
	6	9	18.37%	20	15.75%	1	14.29%	0	0.00%
	Total	49	100%	127	100%	7	100%	4	100%
Male	1	6	13.04%	7	6.73%	1	9.09%	0	0.00%
	2	6	13.04%	17	16.35%	1	9.09%	1	14.29%
	3	3	6.52%	19	18.27%	4	36.36%	4	57.14%
	4	11	23.91%	20	19.23%	1	9.09%	2	28.57%
	5	11	23.91%	27	25.96%	2	18.18%	0	0.00%
	6	9	19.57%	14	13.46%	2	18.18%	0	0.00%
	Total	46	100%	104	100%	11	100%	7	100%

**Table 4:** The T-score equivalent for MCH-FS overall raw score.

Raw Score	T-Score Ranges	Interpretation
46–52	61–65	mild difficulties
53–58	66–65	moderate difficulties
>59	>70	high difficulties

**Table 5:** Feeding the baby.

Variable		Girl		Boy	
		N	%	N	%
How to feed after pregnancy	Breastfeed	96	47.1%	108	52.9%
	Modified milk	28	63.6%	16	36.4%
	Mixing	53	54.6%	44	45.4%
Course of feeding	The child sought food and ate eagerly.	144	50.4%	142	49.7%
	The child did not seek food but ate willingly.	13	48.2%	14	51.9%
	The baby had to be encouraged to feed.	20	62.5%	12	37.5%
One month of dietary expansion	4–5 months	46	45.5%	55	54.5%
	6–7 months	120	53.6%	104	46.4%
	8–9 months	8	53.3%	7	46.7%
	10–12 months	3	42.9%	4	57.1%
	Still later	0	0.0%	1	100.0%
Dietary expansion methods	Traditionally	69	48.6%	73	51.4%
	BLW	15	48.4%	16	51.6%
	Mixing	93	54.1%	79	45.9%
Parents’ feeding styles	Responsive	128	48.3%	137	51.7%
	Controlling	24	63.2%	14	36.8%
	Indulgent	25	65.8%	13	34.2%
	Uninvolved	0	0.0%	4	100.0%

**Table 6:** Regression analysis of the variables.

Variables	Regression (B)	*p*-Value
Age	1.2	0.01
Gender	−2.5	0.03
Dietary patterns	−0.8	0.05
Physical activity	−0.5	-
Parents’ preferences	−1.0	0.02
Parents’ level of education	−0.3	-
Region of residence (urban/suburban)	1.5	0.01
Infant feeding (breast/modified milk)	−1.0	0.03

## Data Availability

Data are contained within the article.
